# Chemotherapy combined with lenvatinib and PD-1 may be a potential better alternative option for advanced unresectable intrahepatic cholangiocarcinoma: a retrospective real-world study

**DOI:** 10.3389/fimmu.2024.1463574

**Published:** 2024-09-03

**Authors:** Zhitao Dong, Chengjun Sui, Jiongjiong Lu, Junwu Guo, Kecai Duan, Kui Wang, Li Geng, Binghua Dai, Jiamei Yang

**Affiliations:** ^1^ Department of Special Treatment, Shanghai Eastern Hepatobiliary Surgery Hospital, Shang Hai, China; ^2^ Department of Hepatic Surgery, Shanghai Eastern Hepatobiliary Surgery Hospital, Shang Hai, China

**Keywords:** unresectable ICC, programmed cell death protein 1 (PD-1), tyrosine kinase, PD-1 inhibitor, systematic therapy

## Abstract

**Background:**

Currently, the prognosis of advanced intrahepatic cholangiocarcinoma (ICC) is poor, and the current treatment methods are not effective.

**Objective:**

The aim of this study was to evaluate the anticancer efficacy of chemotherapy combined with PD-1 inhibitors and tyrosine kinase inhibitors (TKIs) in patients with ICC.

**Methods:**

We retrospectively screened patients with advanced intrahepatic cholangiocarcinoma (ICC) who received chemotherapy combined with lenvatinib and PD-1. We evaluated overall survival (OS), progression-free survival (PFS), the objective response rate (ORR), the disease control rate (DCR), the tumor shrinkage rate, and safety.

**Results:**

We enrolled 95 patients with ICC and divided them into three groups with a median follow-up duration of 15.1 months. The chemotherapy group (chemo-regimen group), chemotherapy combined with immune checkpoint inhibitors (dual-regimen group), and chemotherapy combined with lenvatinib (triple-regimen group) had median OS times of 13.1 months, 20.8 months, and 39.6 months, respectively. Notably, the triple-regimen group had a significantly longer OS than did the chemo-regimen and dual-regimen groups. The chemo-regimen group, dual-regimen group, and triple-regimen group reported median PFS durations of 4.8 months, 11.9 months, and 23.4 months, respectively. Both combination groups exhibited significantly longer PFS than the chemotherapy-only group (P<0.05). The ORRs of the chemo-regimen, dual-regimen, and triple-regimen groups were 18.2%, 55.5%, and 54.7%, respectively. The DCRs were 72.7%, 90%, and 96.2%, respectively, indicating significantly better outcomes in the combination therapy groups.

**Conclusion:**

The combination of chemotherapy with PD-1 inhibitors and lenvatinib demonstrates considerable efficacy and tolerability as a treatment strategy for patients with advanced ICC.

## 1 Introduction

Gemcitabine plus cisplatin (GC) therapy has been established as the first-line treatment for advanced ICC; however, the objective response rate (ORR) remains relatively low. The ABC-002 study reported an ORR of 21%-37% for biliary tract cancer (BTC) (1). The median overall survival (OS) was 11.7 months, whereas it was 8.1 months in the control group (OR=0.64; 95% CI=0.52-0.80, P<0.001). The median progression-free survival (PFS) in the GC group was 8.0 months, whereas it was 5.0 months in the control group. Furthermore, the ABC-06 study demonstrated that FOLFOX chemotherapy (a regimen of folinic acid (2), fluorouracil, and oxaliplatin) marginally improved overall survival (OS) in patients with advanced BTC compared with active symptom control (6.2 months vs. 5.3 months). The efficacy of chemotherapy is notably limited, and alternative treatment options are scarce, particularly after the development of resistance or disease progression.

Immune checkpoint inhibitors (ICIs) have shown some degree of clinical efficacy in liver cancer; however, the effectiveness of single-agent immunotherapy is often constrained by the high heterogeneity and immunosuppressive nature of the TME (3). An emerging strategy involves combining PD-1/PD-L1 inhibitors with antiangiogenic drugs or chemotherapies that possess immunomodulatory properties to counteract TME immunosuppression. This approach is superior to standard treatments. For example, a single-arm phase II clinical trial demonstrated that the combination of lenvatinib and pembrolizumab induced a tumor response (4), with an ORR of 25% in advanced BTC patients and a median progression-free survival (PFS) and OS of 4.9 months and 11.0 months, respectively. A similar trial evaluating camrelizumab combined with GEMOX reported an ORR of 54%, with a median PFS and OS of 6.1 months and 11.8 months, respectively (5).

The integration of ICIs with antiangiogenic drugs and chemotherapy has led to significant advancements in the treatment of advanced BTC. A notable phase II clinical trial conducted by Shi et al. included 30 patients with pathologically confirmed advanced ICC. These patients received first-line treatment comprising Gemox chemotherapy combined with anti-PD-1 antibodies and lenvatinib (6). The outcomes of this trial were promising, with a median PFS of 10.0 months, a median OS that was not reached, and an ORR of 80%. Similarly, Li et al. reported the efficacy of tislelizumab combined with lenvatinib and the Gemox regimen as conversion therapy for potentially resectable locally advanced BTC, yielding an ORR of 56% and a disease control rate (DCR) of 92%. These studies underscore the potential of combining immunotherapy with targeted therapy and systemic chemotherapy as a viable and effective treatment approach for advanced BTC characterized by favorable ORRs.

Therefore, we conducted a retrospective study using preclinical data to assess the safety and efficacy of lenvatinib in conjunction with PD-1 inhibitors and chemotherapy regimens in a real-world setting in patients with advanced ICC.

## 2 Methods

### 2.1 Participants

In this study, we enrolled consecutive patients who presented to Shanghai Eastern Hepatobiliary Surgery Hospital between February 2019 and October 2022. Eligible patients were diagnosed with advanced ICC on the basis of imaging data, including computed tomography (CT), magnetic resonance imaging (MRI), and magnetic resonance cholangiopancreatography (MRCP), in conjunction with pathological biopsy. Biopsy methods included cytological sampling of the perihilar cholangiocarcinoma by brushing or endoscopic retrograde cholangiopancreatography.

For inclusion in the study, patients had to meet specific criteria as per the Response Evaluation Criteria in Solid Tumors (RECIST) version 1.1, which necessitated the presence of at least one measurable lesion. Patients who had previously received treatment were excluded from this study.

The criteria for diagnosing advanced ICC were as follows (7, 8): (1) biopsy indicative of poor differentiation, (2) evidence of portal vein or inferior vena cava invasion, and (3) multiple lymph nodes or distant metastases confirmed by imaging.

### 2.2 Inclusion criteria

Eligible participants were required to have an Eastern Cooperative Oncology Group performance status (ECOG PS) of 0 or 1, a life expectancy of at least one month, and at least one measurable lesion, as defined by RECIST 1.1. Additionally, patients were required to have a Child−Pugh grade of A or B. For patients presenting with obstructive jaundice, initial biliary drainage was performed to ensure the safety of the subsequent treatment regimen.

### 2.3 Exclusion criteria

Patients with a history of prior treatments such as Transarterial Chemoembolization (TACE), radiation therapy, ablation, or Hepatic Arterial Infusion Chemotherapy (HAIC) were excluded. Similarly, those who had received PD-1, PD-L1, or MEK inhibitors, as well as those with a history of autoimmune diseases or other malignancies, did not meet the study’s inclusion criteria. The study also excluded patients lacking the comprehensive imaging data required for accurate tumor response evaluation. Furthermore, patients who were lost to follow-up or had uncontrolled intercurrent illnesses were also excluded.

### 2.4 Ethical considerations and patient consent

This study was conducted in strict accordance with the principles of the Declaration of Helsinki and the International Conference on Harmonization Good Clinical Practice guidelines. This study was approved by the Ethics Committee of Eastern Hepatobiliary Hospital (ethics code: EHBHKY2019-K-027.1/3/2020). Before the commencement of treatment, informed consent was obtained from all participants, and their data were anonymized for clinical research. The confidentiality and anonymity of the patients’ information were rigorously maintained, ensuring that patient identities were not discernible in any reports or publications. This report aligns with the Strengthening the Reporting of Observational Studies in Epidemiology (STROBE) statement.

### 2.5 Treatment regimen

In this study, the participants were stratified into three distinct treatment groups, each receiving a different therapeutic regimen.

#### 2.5.1 Triple-regimen group (chemo+ICI+TKI)

This group received a combination of lenvatinib, PD-1 inhibitors, and chemotherapy. The lenvatinib dosage was determined on the basis of body weight: patients weighing ≥60 kg received 12 mg of lenvatinib orally once daily, whereas those weighing <60 kg received 8 mg of lenvatinib. PD-1 inhibitor therapy involved a fixed dose of 200 mg administered every three weeks, with three different PD-1 drugs available (tislelizumab, toripalimab, and sintilimab). The chemotherapy regimens included gemcitabine plus cisplatin (GC) administered intravenously every three weeks for a total of six cycles. Following the completion of chemotherapy, immunotherapy and targeted therapy were continued until the disease progressed.

#### 2.5.2 Dual-regimen group (chemo+ICI)

Patients in this group received a combination of a GC regimen and PD-1 inhibitors. The chemotherapy regimens used were similar to those used in the three-drug combination group.

#### 2.5.3 Control group (chemo)

This group was treated with a GC regimen.

The treatment continued until disease progression or unacceptable toxicity occurred or some other conditions were judged by the investigator as inappropriate for continuing the treatment. Once severe toxicity occurred, the administration would be delayed and/or the dose would be reduced according to the drug’s instructions.

### 2.6 Evaluation methods

The tumor response in this study was meticulously evaluated by a panel of three experienced radiologists using RECIST version 1.1 and mRECIST criteria. Imaging assessments, predominantly conducted via MRI (or CT when MRI was unavailable), were performed at baseline and subsequently every 4–8 weeks following each treatment cycle. Radiological assessments were performed at least four weeks after the initial observation to confirm complete remission (CR) and partial remission (PR).

Adverse events (AEs) were comprehensively documented from the commencement of treatment until one month after its conclusion. These events were classified and graded according to the National Cancer Institute Common Terminology Criteria for Adverse Events (CTCAE), version 4.03.

### 2.7 Treatment endpoints

The primary endpoints defined for this study were PFS and OS. The secondary endpoints included the ORR and DCR, along with safety evaluations. The ORR was defined as the aggregate percentage of patients who achieved CR or PR. Both the ORR and DCR were calculated on the basis of the standards set by RECIST 1.1 and mRECIST, respectively.

### 2.8 Statistical analysis

The data are described herein as means ± standard errors for normally distributed values and as medians (interquartile ranges [IQRs]) for nonnormally distributed values. Categorical variables of the baseline characteristics are presented as numbers (n) and ratios (%). The Wilcoxon rank-sum test, Student’s t test, Pearson’s chi-square test, and Fisher’s exact test were used as appropriate to compare baseline characteristics between groups. The median PFS and OS rates and 95% confidence intervals (CIs) in the total population and subgroups were estimated by using the Kaplan–Meier method, and the log-rank test was used to analyze the differences in the survival curves. Unadjusted hazard ratios (HRs) were initially derived via Cox regression without including covariates or propensity scores in the model. The ORR was calculated as a percentage with two-sided 95% confidence intervals (CIs) via the Clopper–Pearson method. Programming and statistical analyses were performed with SPSS (version 20.0). All the statistical analyses were two-sided, and p values less than 0.05 were considered to indicate statistical significance.

## 3 Results

### 3.1 General characteristics of the patients

**Figure 1:**
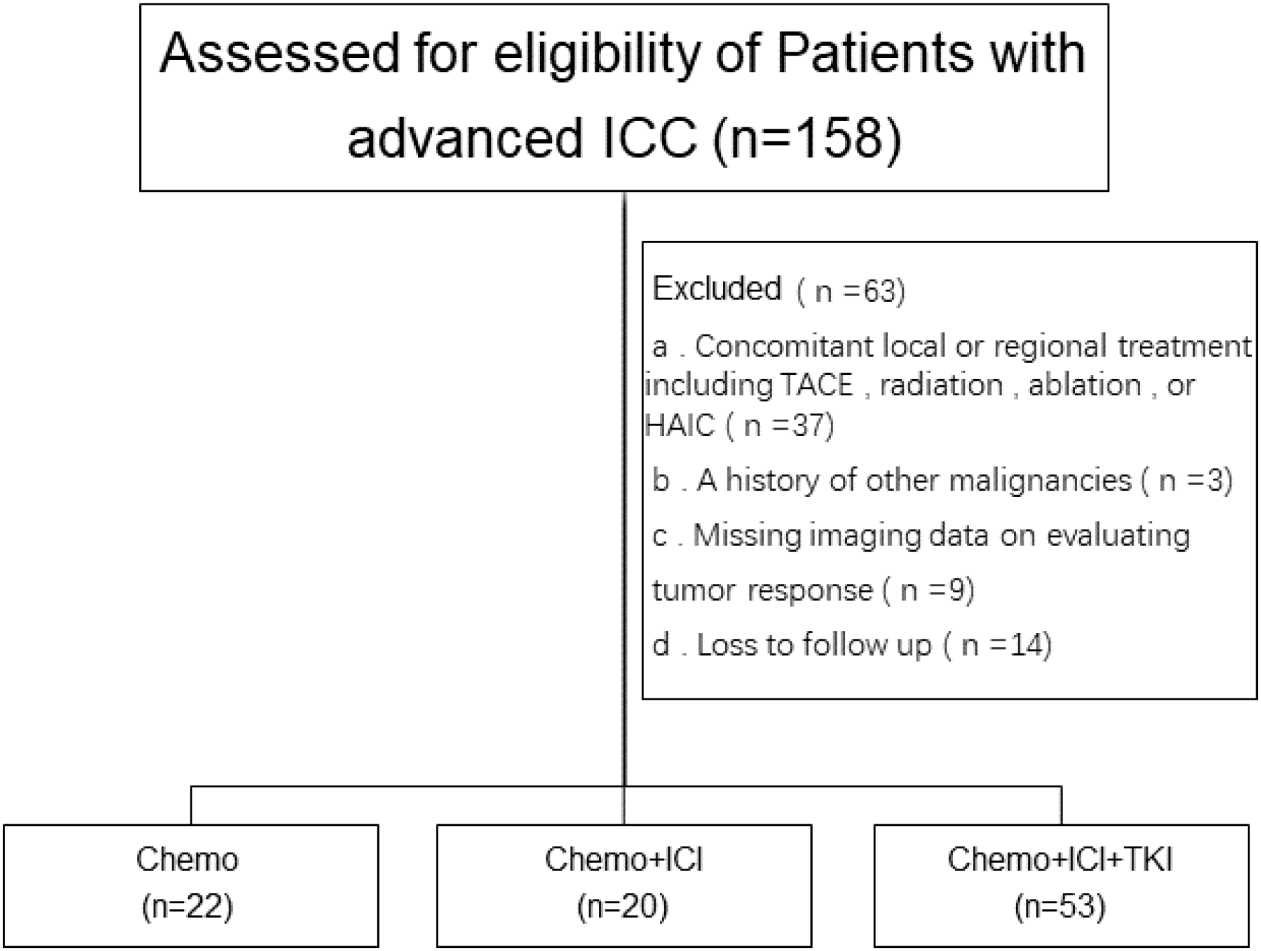
The flow chart of the study illustrates the enrollment procedure.

Among the initial pool of 158 patients screened for this study, a subset of patients was excluded on the basis of the following criteria: 37 patients had undergone transarterial chemoembolization (TACE), radiofrequency ablation, or hepatic arterial infusion (HAIC) chemotherapy; 3 patients had a history of other malignant tumors; 9 patients lacked complete imaging data; and 14 patients were lost to follow-up. A research flowchart is shown in **Figure 1**.

Consequently, 95 patients were enrolled in this study. Among the 95 enrolled patients, 22 received chemotherapy (chemotherapy group), 20 received a chemotherapy regimen plus PD-1 inhibitor treatment (dual-combination group), and 53 received a chemotherapy regimen plus PD-1 inhibitor plus lenvatinib treatment. The PD-1 inhibitors predominantly included tislelizumab, toripalimab, and sintilimab.

### 3.2 Baseline characteristics

**Table 1** presents a detailed summary of the demographic and baseline characteristics of all the enrolled patients. The median patient age at the initiation of treatment was 58 years. Most patients were male, constituting 64.2% of the cohort (61/95), whereas 25.8% were female (34/95).

**Table 1:** Baseline demographic and clinical characteristics (n=95).

Character	Factor	Chemo (N=22)	Chemo+ICI (N=20)	Chemo+ICI+TKI (N=53)	Overall (N=95)	P-value
Sex	Male	13 (59.1%)	10 (50.0%)	38 (71.7%)	61 (64.2%)	0.1919
	Female	9 (40.9%)	10 (50.0%)	15 (28.3%)	34 (35.8%)	
Age	Mean / Std	59.9 / 11.20	56.5 / 8.99	58.4 / 8.98	58.3 / 9.50	0.5792
	Median	62	57	57	58	
	Inter Quartile Range	52, 69	51, 60	53, 65	52, 65	
Child-Pugh stage	A	22 (100%)	19 (95.0%)	53 (100%)	94 (98.9%)	0.1503
	B	0	1 (5.0%)	0	1 (1.1%)	
Lymph node	No	13 (59.1%)	11 (55.0%)	34 (64.2%)	58 (61.1%)	0.7567
	Yes	9 (40.9%)	9 (45.0%)	19 (35.8%)	37 (38.9%)	
Metastasis	No	14 (63.6%)	12 (60.0%)	38 (71.7%)	64 (67.4%)	0.5812
	Yes	8 (36.4%)	8 (40.0%)	15 (28.3%)	31 (32.6%)	
Maximum tumor diameter	Mean / Std	80.373 / 46.8786	83.099 / 42.6172	81.600 / 49.4813	81.631 / 47.0460	0.9629
	Median	77.00	76.35	75.00	75.00	
	Inter Quartile Range	46.00, 102.00	49.35, 119.94	54.00, 96.92	52.70, 102.00	
HBV	Positive	16 (72.7%)	8 (40.0%)	19 (35.8%)	43 (45.3%)	0.0143
	Negative	6 (27.3%)	12 (60.0%)	34 (64.2%)	52 (54.7%)	
Hepatolithiasis	No	0	1 (5.0%)	1 (1.9%)	2 (2.1%)	0.5224
	Yes	22 (100%)	19 (95.0%)	52 (98.1%)	93 (97.9%)	
Diabetes	No	20 (90.9%)	17 (85.0%)	43 (81.1%)	80 (84.2%)	0.5685
	Yes	2 (9.1%)	3 (15.0%)	10 (18.9%)	15 (15.8%)	
Hypertension	No	17 (77.3%)	17 (85.0%)	45 (84.9%)	79 (83.2%)	0.7018
	Yes	5 (22.7%)	3 (15.0%)	8 (15.1%)	16 (16.8%)	
Differentiated degree	Low differentiation	10 (45.5%)	9 (45.0%)	10 (18.9%)	29 (30.5%)	0.0771
	Middle to low differentiation	3 (13.6%)	4 (20.0%)	17 (32.1%)	24 (25.3%)	
	moderately differentiated	9 (40.9%)	7 (35.0%)	26 (49.1%)	42 (44.2%)	
Tumor number	=1	12 (54.5%)	11 (55.0%)	22 (41.5%)	45 (47.4%)	0.2070
	≥2	6 (27.3%)	9 (45.0%)	29 (54.7%)	44 (46.3%)	
CA199	<37	5 (22.7%)	7 (35.0%)	23 (43.4%)	35 (36.8%)	0.1430
	≥37	17 (77.3%)	13 (65.0%)	30 (56.6%)	60 (63.2%)	
NLR	Mean / Std	4.59/3.69	3.49 / 1.90	4.57 / 3.15	4.35 / 3.07	0.3728
	Median	3.72	2.77	3.66	3.35	
	Inter Quartile Range	2.26, 5.71	2.24, 4.58	2.49, 5.99	2.36, 5.71	
PLR	Mean / Std	195.93 / 123.16	177.21 / 75.39	161.61 / 90.30	172.96/ 96.22	0.3690
	Median	154.16	178.75	146.03	152.34	
	Inter Quartile Range	115.08, 229.93	112.25, 228.41	101.53, 222.73	104.73, 227.47	

NLR, Neutrophil-to-Lymphocyte Ratio; PLR, Platelet-to-Lymphocyte Ratio.

A significant majority of patients (94.7% (90/95) had an Eastern Cooperative Oncology Group (ECOG) performance status of 0-1), indicating relatively good physical functioning. Nearly all patients (98.9%, 94/95) were classified as Child−Pugh stage A, reflecting relatively preserved liver function. At baseline, 63.2% of the patients (60/95) had abnormal levels of the tumor antigen CA19-9. Furthermore, 45.3% of patients (43 of 95) had a history of hepatitis B infection. A total of 30.5% (29/87) had a poorly differentiated histology, 24 patients had a well-differentiated histology, and 42 patients had a moderately differentiated histology. Prior to treatment, 31 patients had distant metastases, predominantly in the lungs (22 patients, 26.3%), bones (five patients, 5.26%), and brain (two patients, 2.10%). There were also multiple organ metastases (two patients, 2.10%). Thirty-seven patients (38.9%, 30/95) had positive lymph nodes at the beginning of the study.

### 3.3 Efficacy

#### 3.3.1 The median treatment duration

The median treatment duration across the three study groups was 8.0 months, with an IQR of 5.7 to 12.0 months. This duration varied among the groups; patients in the chemo-regimen group underwent a median of 4.0 chemotherapy cycles (IQR 3.0–6.0). In the dual-regimen group, patients received a median of five treatment cycles (IQR 4.0–8.0). Patients in the triple-regimen group had a median of six treatment cycles (IQR 3.0–9.0) (**Table 2**).

**Table 2:** Confirmed anti-tumour activity (evaluated by modified RECIST).

	Chemo (n=22)	Chemo+ICI (n=20)	Chemo+ICI+TKI (n=53)
Tumor Response			
Complete response	0% (0)	10% (2)	9.4% (5)
Partial response	18.2% (4)	45% (9)	47.2% (25)
Stable disease	31.8% (7)	40% (8)	39.6% (21)
Progressive disease	50% (11)	5% (1)	3.8% (2)
Objective Response	18.2% (4/22)	55.0% (11/20)	56.6% (30/53)
Disease Control Rate	50% (11/22)	95% (19/20)	96.2% (51/53)
Median duration of response (months)	8.9m (IQR:5.50-14.03)	10.7 m (IQR:9.05-15.85)	14.2 m (IQR:10.65-25.15)
Tumour Shrinkage Duration			
< 6 months	1	1	1
≥ 6 months	1	6	9
≥ 12 months	2	3	19
Median treatment duration (Cycles)	4 (IQR:3-6)	5 (IQR:4-8)	6 (IQR:3-9)
Median treatment duration (Months)	8.0 (IQR:5.7-12.0)		
Median follow-up duration (Months)	36.6 (IQR: 34.4-38.7)	32.6 (IQR: 30.2-34.9)	33.1 (IQR: 30.8-35.3)

#### 3.3.2 Follow-up duration

The median follow-up duration for the chemo-regimen group was 36.6 months (IQR: 34.4-38.7), that for the dual-regimen group was 32.6 months (IQR: 30.2-34.9), and that for the triple-regimen group was 33.1 months (IQR: 30.8-35.3). At the last follow-up, ten patients in the triple-therapy group did not exhibit disease progression and continued maintenance-targeted immunotherapy. There were no significant differences in the follow-up times among the three groups (P > 0.05) (**Table 2**).

#### 3.3.3 Overall survival (OS)

**Figure 2:**
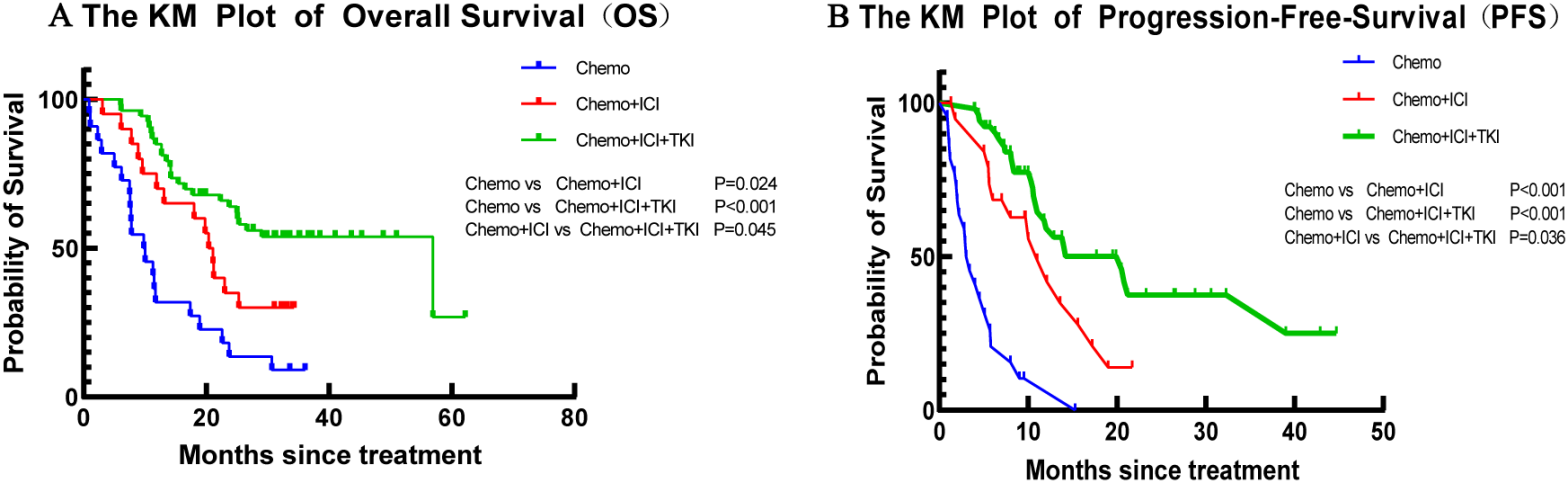
The Kaplan–Meier curves of **(A)** OS and **(B)** PFS in patients of the Chemo group, the Dual-regimen group, and the Triple group, respectively.

In the present study, the median OS varied across the treatment groups. For the group that received chemotherapy alone, the median OS was 13.1 months, with an IQR of 8.8–17.5 months. In the dual-regimen group, the median OS was 20.8 months (IQR: 16.1–25.4). The triple-regimen group had a further extended median OS of 39.6 months (IQR: 33.2 to 45.9) (**Figure 2A**). The dual-regimen and triple-regimen groups had significantly different OS rates (chemo-regimen group vs. dual-regimen group, P=0.024; dual-regimen group vs. triple-regimen group, P=0.045; chemo-regimen group vs. triple-regimen group, P<0.001).

#### 3.3.4 Progression-free survival (PFS)

The median PFS time for patients who received chemotherapy alone was 4.8 months (IQR 3.0–6.7 months) (**Figure 2B**). In the dual-regimen group, the median PFS time was 11.9 months (IQR 9.0–14.8 months). The median PFS of patients in the triple-regimen group was slightly greater at 23.4 months (IQR 18.2–28.7 months). Statistical analysis revealed significant differences in PFS between the chemotherapy group and the other two therapy groups (chemo-regimen group vs. dual-regimen group, P <0.001; dual-regimen group vs. triple-regimen group, P <0.001; chemo-regimen group vs. triple-regimen group, P=0.036).

#### 3.3.5 Optimal response time

**Figure 3:**
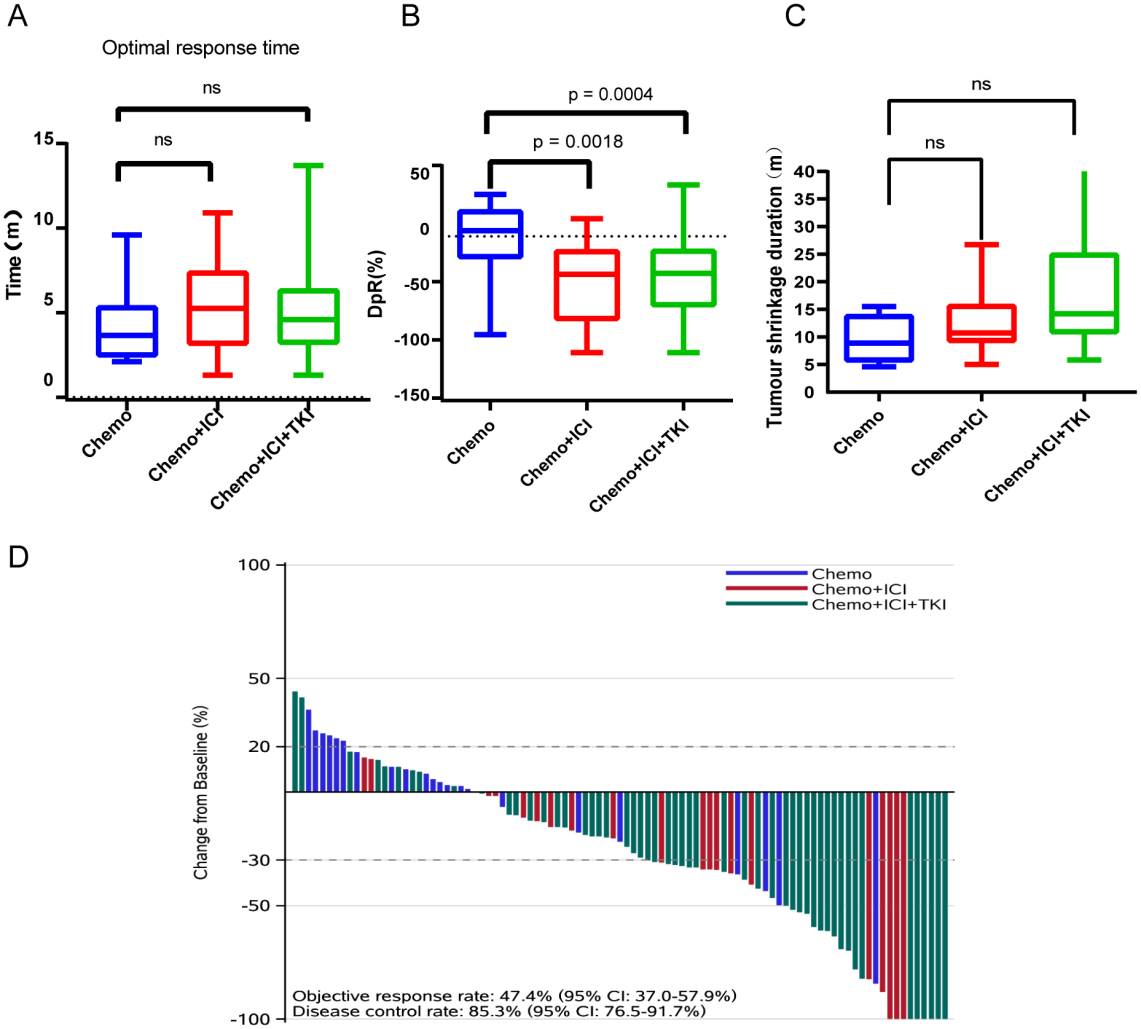
Analysis of optimal response time **(A)**, average tumor shrinkage depth (DpR) **(B)**, tumor shrinkage duration **(C)**, and treatment effect **(D)**. NS: not significant.

The median response time to tumor treatment in the chemo-regimen group was 3.65 months, with an IQR of 2.40–5.40 months (**Figure 3A**). Patients in the dual-regimen group had a median response time of 5.25 months, with an IQR of 3.075–7.45 months. The median response time in the triple-regimen group was 4.60 months, with an IQR of 3.15 to 6.40 months. Statistical analysis revealed no significant differences in the median response times between the chemo-regimen and triple-regimen groups (P=0.5281). Similarly, no significant differences were detected between the chemo-regimen and dual-regimen groups or between the dual-regimen and triple-regimen groups (P=0.3652 and P=0.5049, respectively).

#### 3.3.6 Early tumor regression rate (early tumor shrinkage, ETS)

This study characterized early tumor shrinkage (ETS) as tumor regression of ≥20% after 6–8 weeks of treatment initiation. The median ETS rates observed in the chemo-, dual-, and triple-regimen treatment groups were 24%, 61%, and 63%, respectively. Comparative analysis indicated that the dual regimen and triple regimens yielded significantly higher ETS rates than the chemotherapy regimen did (P<0.05). However, no significant differences were observed between the dual-regimen group and the triple-regimen group (P=0.3652).

#### 3.3.7 Average tumor shrinkage depth (DpR)

The average tumor shrinkage depths in the chemo-regimen, dual-regimen, and triple-regimen groups were -1.676% (IQR: -18.86 ~ -22.78%), -36.55% (IQR: -72.06 ~ -11.62%), and 34.22% (IQR: -60.21 ~ -11.33%), respectively (**Figure 3B**). A statistically significant difference was observed between the patients who received chemotherapy alone and those who received chemotherapy and ICI therapy (p = 0.0018). There was also a statistically significant difference between the chemo- and triple-regimen groups, as indicated by a P value of 0.0004. However, no statistically significant difference was found between the dual- and triple-regimen groups, as evidenced by a P value of 0.8109.

#### 3.3.8 DpR duration (months)/tumor shrinkage duration 

The median durations of tumor shrinkage in the chemo-regimen, Dual-regimen, and triple-regimen groups were 8.9 months (IQR: 5.50–14.03), 10.7 months (IQR: 9.05–15.85), and 14.2 months (IQR: 10.65–25.15), respectively (**Figure 3C**). Statistical analysis revealed no significant differences in the duration of tumor shrinkage among the three groups. The P values for the comparisons were as follows: between the chemo-regimen group and the dual-regimen group, 0.39; between the chemo-regimen group and the triple-regimen group, 0.10; and between the dual-regimen group and the triple-regimen group, 0.11.

#### 3.3.9 Treatment effect

**Figure 4:**
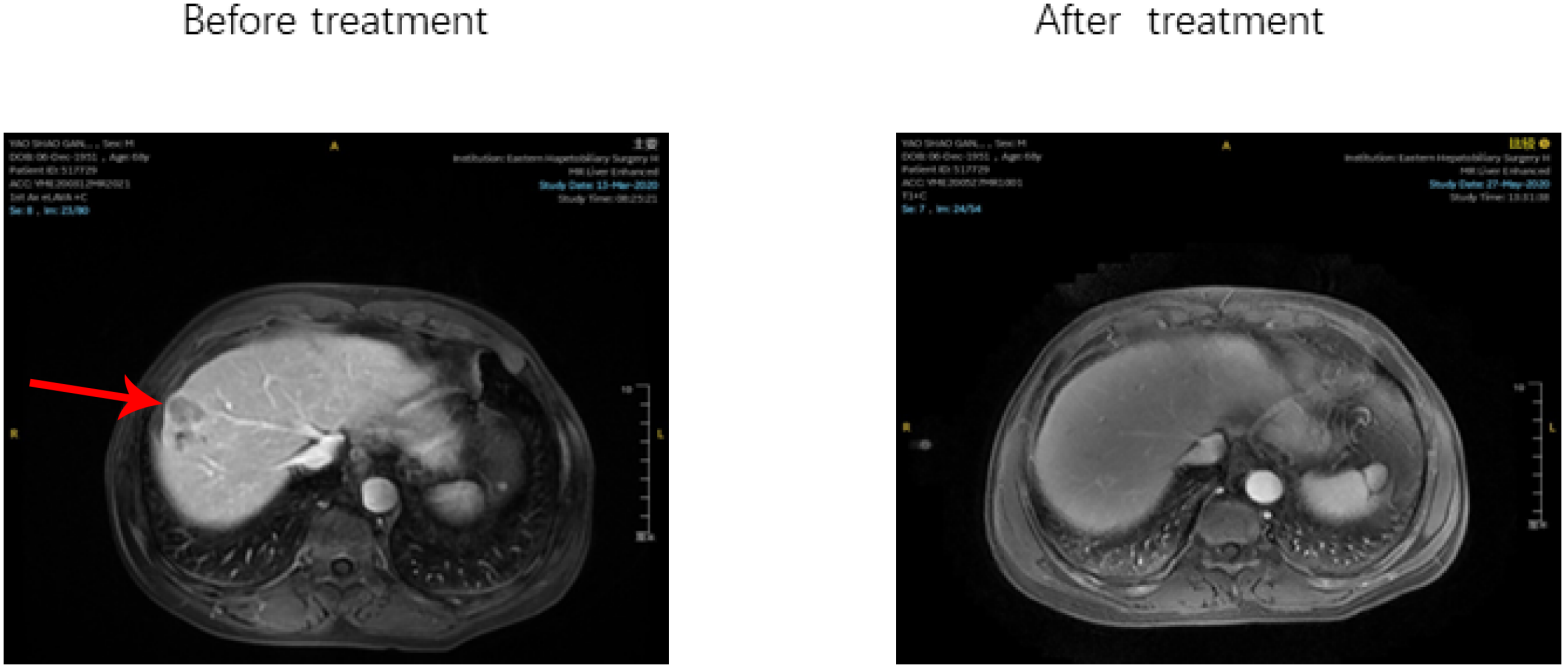
After receiving Triple regimen treatment, the patient's lesion completely disappeared.

After treatment, the patients’ overall DCR was reported to be 89.5%, with an IQR of 82.7–95.9%. The ORR for the entire cohort was 46.3%, with an IQR of 37.4%–59.2%. The ORRs of patients in the chemo-regimen, dual-regimen, and triple-regimen groups were 18.2% (4/22), 55.5% (11/20), and 54.7% (29/53), respectively. The DCRs of the three groups were 72.7% (16/22), 90% (18/20), and 96.2% (51/53), respectively (**Figure 3D**; **Table 2**). In the dual-regimen group, 10.0% of the patients achieved CR, whereas 9.4% of the patients reached CR in the triple-regimen group (**Figure 4**).

After treatment, the observed response duration varied across the three treatment groups. In the chemo-regimen group, 75% of the patients had a response duration exceeding six months, and 50% experienced a response lasting more than one year. In the dual-regimen group, 90.0% of the patients sustained a response for more than half a year, and 30% had a response duration extending beyond one year. The triple-regimen group included a majority (96.6%) of patients with response durations exceeding six months, and 65.5% of patients in this group experienced responses lasting more than one year.

**Figure 5:**
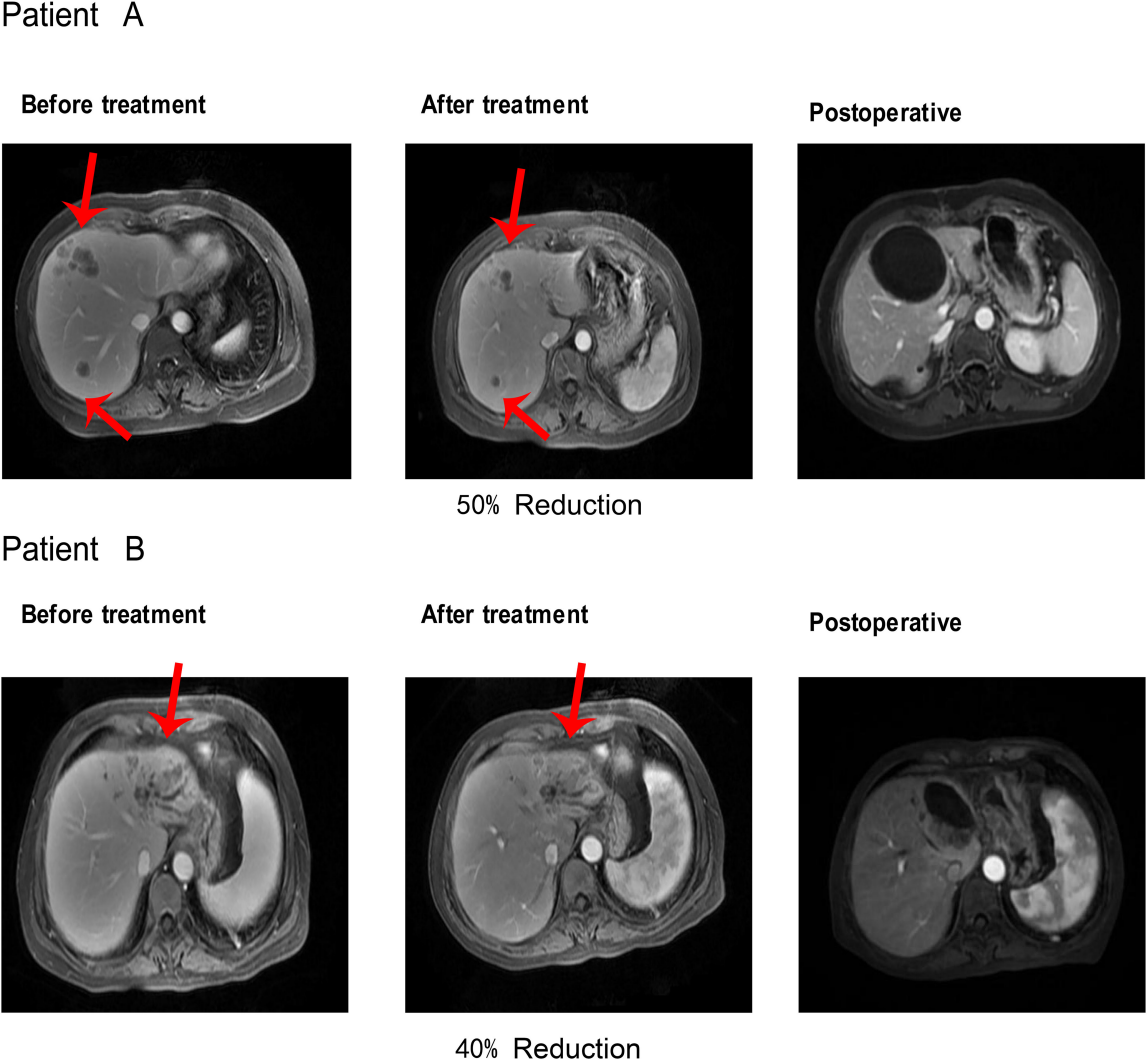
After receiving Triple regimen treatment, patients A and B significantly reduced lesion size and underwent radical surgical resection.

Within the triple-regimen group, two patients exhibited notable posttreatment outcomes, enabling them to undergo radical surgical intervention (**Figure 5**).

The first patient achieved a 50% reduction in tumor size after five cycles of the combined treatment regimen, which was classified as a partial response (PR) according to the mRECIST criteria. This significant shrinkage allowed for successful radical resection of the liver tumor, after which the patient was discharged. Similarly, the second patient completed six cycles of combined treatment, resulting in a 40% reduction in tumor size, and was also deemed a PR according to the mRECIST criteria. This reduction facilitated radical resection of the liver tumor, followed by subsequent discharge. These instances highlight the efficacy of the triple-therapy regimen in significantly reducing tumor size, thereby making patients eligible for potentially curative surgical procedures.

#### 3.3.10 Subgroup analysis

**Figure 6:**
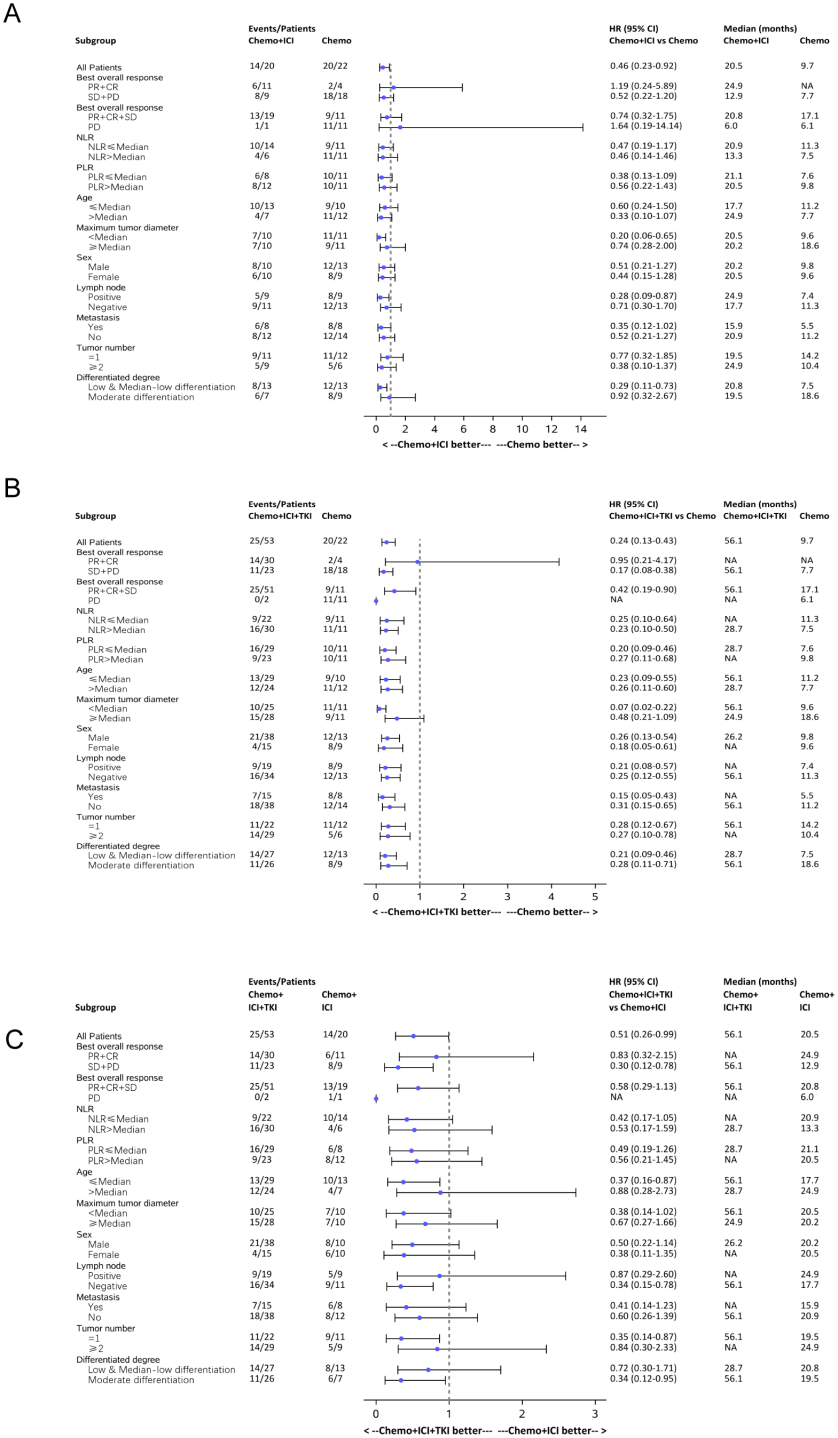
Forest plot analysis of subgroups of PFS in all the three group patients. **(A)** Forest plot analysis of subgroups of PFS between the chemo-regimen and dual-regimen group. **(B)** Forest plot analysis of subgroups of PFS between the chemo-regimen and triple-regimen group. **(C)** Forest plot analysis of subgroups of PFS between the dual-regimen and triple-regimen group.

**Figure 7:**
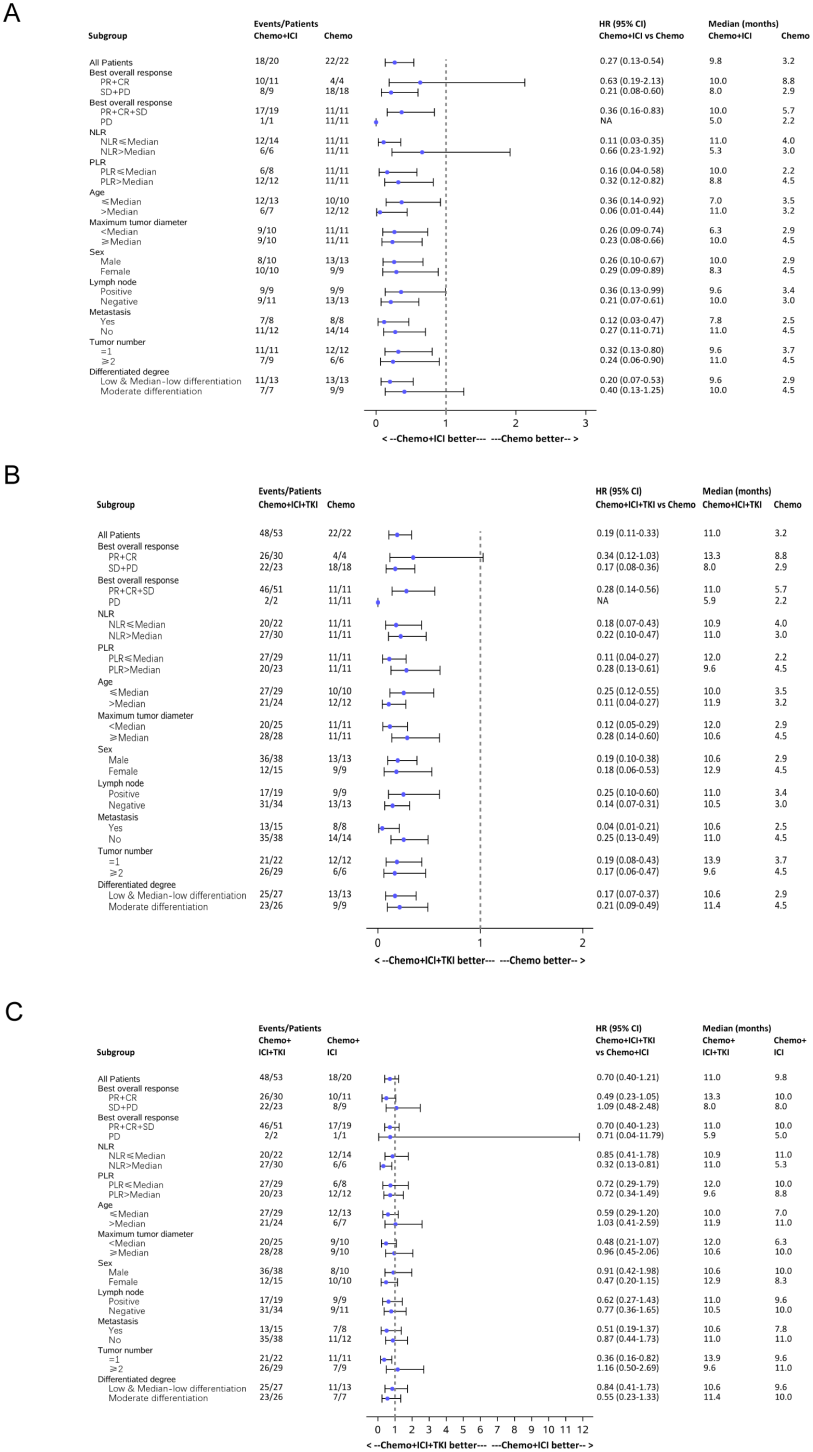
Forest plot analysis of subgroups of OS in all the three group patients. **(A)** Forest plot analysis of subgroups of OS between the chemo-regimen and dual-regimen group. **(B)** Forest plot analysis of subgroups of OS between the chemo-regimen and triple-regimen group. **(C)** Forest plot analysis of subgroups of OS between the dual-regimen and triple-regimen group.

A comprehensive subgroup analysis was conducted to compare the survival outcomes among the three patient groups, with a focus on the median PFS and median OS across different stratifications, as illustrated in the forest plot (**Figures 6**, **7**). The three groups were compared and analyzed, but no clear high-risk factors were found.

#### 3.3.11 Safety analysis

Throughout the treatment in this study, all 95 patients (representing 100% of the cohort) reported experiencing AEs, but notably, there were no instances of grade 5 AEs (**Table 3**). The incidence and nature of Grade ≥ 3 tumors varied across different treatment groups. In the chemo-regimen group, 22.7% (5/22) of the patients had Grade 3 or higher AEs, predominantly involving myelosuppression. Thirty percent (6/20) of the patients in the dual-regimen group AEs of similar severity, with palmoplantar erythema being the most common. In the triple-regimen group, which received combined chemotherapy, immune checkpoint inhibitors, and targeted therapy, 32.1% (17/53) of the patients experienced grade 3 or higher AEs, mainly palmoplantar erythema and pneumonia (**Table 3**). Nevertheless, the AEs in the combination therapy groups were generally safe, well tolerated, and manageable.

**Table 3:** Summary of the TRAEs in patients (n=95).

	Complications											
	Chemo(n=22 )				Chemo+ICI(n=20)				Chemo+ICI+TKI(n=53)			
	<3		≥3		<3		≥3		<3		≥3	
Weakness	2	9.09%	0	0.00%	2	10.00%	0	0.00%	4	7.55%	0	0.00%
Decreased Appetite	3	13.64%	0	0.00%	3	15.00%	0	0.00%	5	9.43%	0	0.00%
Fever	1	4.55%	0	0.00%	1	5.00%	0	0.00%	2	3.77%	0	0.00%
Rash	2	9.09%	0	0.00%	2	10.00%	1	5.00%	6	11.32%	2	3.77%
Palmar And Plantar Erythema	1	4.55%	0	0.00%	2	10.00%	2	10.00%	8	15.09%	4	7.55%
Elevated ALT Or AST	1	4.55%	0	0.00%	1	5.00%	0	0.00%	2	3.77%	0	0.00%
Proteinuria	1	4.55%	0	0.00%	1	5.00%	0	0.00%	3	5.66%	0	0.00%
Anemia	2	9.09%	0	0.00%	1	5.00%	0	0.00%	2	3.77%	0	0.00%
Thrombocytopenia	1	4.55%	2	9.09%	2	10.00%	1	5.00%	3	5.66%	2	3.77%
Abdominal Pain	1	4.55%	0	0.00%	1	5.00%	0	0.00%	1	1.89%	0	0.00%
Hypothyroidism	0	0.00%	0	0.00%	0	0.00%	0	0.00%	3	5.66%	1	1.89%
Pruritus	0	0.00%	0	0.00%	0	0.00%	0	0.00%	4	7.55%	0	0.00%
Elevated Blood Bilirubin	1	4.55%	0	0.00%	1	5.00%	0	0.00%	2	3.77%	0	0.00%
Hypertension	0	0.00%	0	0.00%	1	5.00%	0	0.00%	2	3.77%	1	1.89%
Diarrhea	2	9.09%	1	4.55%	2	10.00%	0	0.00%	5	9.43%	1	1.89%
Nausea	2	9.09%	0	0.00%	2	10.00%	0	0.00%	4	7.55%	0	0.00%
Neutropenia	2	9.09%	2	9.09%	2	10.00%	1	5.00%	3	5.66%	2	3.77%
Vomit	1	4.55%	0	0.00%	1	5.00%	0	0.00%	2	3.77%	0	0.00%
Pneumonia	0	0.00%	0	0.00%	1	5.00%	1	5.00%	5	9.43%	3	5.66%
Myocardial Damage	0	0.00%	0	0.00%	0	0.00%	0	0.00%	2	3.77%	2	3.77%

ALT, alanine aminotransferase; AST, aspartate transaminase; TRAE, treatment-related AE.

## 4 Discussion

### 4.1 Effectiveness analysis

ICC is a highly malignant tumor with a poor prognosis and a low response rate. This is the first study to compare the efficacy and safety of three treatment regimens (chemotherapy alone, chemotherapy+PD-1, and chemotherapy+TKI+PD-1) in real-world patients with advanced ICC. Our results indicated that triple therapy (chemotherapy + TKI + PD-1) as a first-line treatment yielded better PFS and (OS than the other regimens. This approach demonstrated significant antitumor activity in patients with ICC, with notable median PFS and OS rates and high ORRs, DCRs, and CBRs. These findings suggest that a combination of targeted therapy, immunotherapy, and systemic chemotherapy is effective for BTC treatment, which aligns with the outcomes reported in other studies of similar regimens.

The recent advancements in combination therapies for unresectable or advanced malignant biliary tract tumors have been significant (9). The TOPAZ-1 trial (NCT03875235) highlighted the efficacy of combining durvalumab with gemcitabine and cisplatin (chemotherapy regimens) as first-line treatment. As of February 25, 2022, the OS survival rate was 76.9%. The median OS (95% CI) was 12.9 (11.6–14.1) months in the experimental group and 11.3 (10.1–12.5) months in the control group.

These findings reflect the ongoing commitment of researchers to improve therapeutic strategies for challenging biliary tract cancers (10).

Antiangiogenesis targeted therapy inhibits tumor angiogenesis and tumor cell proliferation and improves the tumor immune microenvironment, resulting in a synergistic enhancement mechanism with immunotherapy. In the treatment of advanced liver cancer, the combination of antiangiogenic targeted therapy and immunotherapy has become the preferred first-line treatment strategy. In the treatment of biliary tract tumors, a combination of chemotherapy, immunotherapy, and antiangiogenic targeted therapy has also been actively explored.

In 2020, the European Society for Medical Oncology (ESMO) reported a phase II clinical study conducted in China for locally advanced or metastatic ICC: a combination of toripalimab, lenvatinib, gemcitabine, and the oxaliplatin and gemcitabine (GEMOX) chemotherapy regimen (11), followed by maintenance therapy with toripalimab and lenvatinib after six cycles of treatment. The results revealed that the ORR was as high as 80.0% (24/30), the DCR was 93.3% (28/30), the median PFS was 10.0 months, and the incidence of ≥ grade 3 AEs was 50%. In a phase II randomized controlled study reported by the American Society of Clinical Oncology (ASCO) in 2023 (12), 80 patients with nonsurgically resectable or metastatic BTC were enrolled. The results revealed that the combination therapy group had a significantly longer median PFS (8.6 months vs. 6.2 months, P<0.01), higher ORR (52.8% vs. 29.4%), and median response duration (9.4 months vs. 3.4 months) but also had higher rates of grade 3/4 TRAEs (77.5% vs. 40%).

According to the above studies, the use of chemotherapy combined with immunotherapy and tyrosine kinase inhibitors as antivascular targeted drugs has good prospects, especially with an ORR ranging from 52.8% to 80.0%, indicating the potential for translational therapy. Further phase III clinical studies are needed to confirm its efficacy and safety.

Predicting the efficacy of immunotherapy via biomarkers is also an important direction for research and exploration of BTC immunotherapy. Subgroup analysis of the TOPAZ-1 and KEYNOTE-966 studies suggested that it may not be possible to predict the survival benefit of combined immunotherapy with chemotherapy by dividing patients according to PD-L1 expression. In the TOPAZ-1 study, patients with tumor area positivity (TAP) ≥1% who received combined immunotherapy had an HR of 0.79 (95% CI: 0.61~1.00) for OS, whereas patients with TAP <1% who received combined immunotherapy had an HR of 0.86 (95% CI: 0.60~1.23) for OS. In the KEYNOTE-966 study, patients with a combined positive score (CPS) ≥1 who received combined immunotherapy had an HR of 0.85 (95% CI: 0.72~1.00) for OS, whereas patients with a CPS <1 who received combined immunotherapy had an HR of 0.84 (95% CI: 0.62~1.14) for OS.

### 4.2 Surgical treatment after downstaging with target-free therapy

In this study, two ICC patients achieved significant tumor shrinkage after target-free therapy and were classified as having a PR according to the mRECIST criteria. Both patients underwent conversion therapy, followed by successful radical liver tumor resection. After surgery, the patients recovered well without severe complications, and pathological examinations revealed a 40%-50% tumor shrinkage rate with no residual cancer cells at the tumor margins or lymph nodes. The Multi-Disciplinary Treatment (MDT) team deemed the patients suitable for surgery after downstaging, leading to complete tumor removal and positive postoperative recovery. These cases highlight that a significant treatment response can open up surgical options for ICC patients, emphasizing the need for more clinical studies to explore this approach further.

### 4.3 Evaluation indicators of tumor efficacy

As dynamic methods for assessing tumor treatment efficacy, the early tumor shrinkage rate and depth have significant clinical importance and value. In our study, Groups 2 and 3 presented notably higher early tumor shrinkage rates than did Group 1 (P<0.05). There were also significant differences in the average tumor shrinkage depth between Groups 1 and 2 and between Groups 1 and 3 (P<0.05). These findings suggest that the tumor shrinkage rate and depth are critical indicators of treatment efficacy. Although the triple-regimen group had a longer duration of tumor shrinkage than Groups 2 and 1 did, these differences were not statistically significant (P>0.05). The early tumor shrinkage rate and depth have been reported to correlate positively with OS and PFS in patients. Clinical trials have demonstrated that patients who achieve a significantly early tumor shrinkage rate and depth tend to have better OS and PFS rates. These measures can serve as adjunctive indicators for evaluating treatment efficacy and for enhancing precision and objectivity when used along with traditional static parameters. For example, in metastatic colorectal cancer (mCRC) and non-small cell lung cancer (NSCLC), early tumor shrinkage (ETS) and depth of response (DpR) can stratify patients into distinct subgroups for tailored treatment plans (13). However, the specific values and time points of these indicators vary according to the tumor type and treatment, necessitating further research to standardize and optimize their use.

### 4.4 Mechanism analysis

Chemotherapy enhances the effects of immunotherapy via several mechanisms. First, cytotoxic agents such as platinum and gemcitabine activate apoptosis in monocytes/macrophages, reduce the number of myeloid-derived suppressor cells, and bolster anticancer immunity (14). Second, cytokines from chemotherapy-damaged cells recruit antigen-presenting cells (APCs) (15), facilitating phagocytosis and proinflammatory cytokine secretion by dendritic cells (DCs) (16). Additionally, epigenetic modulators upregulate antigen processing and presentation mechanisms and stimulate cytokine production, further enhancing the immune response (17). Patients resistant to chemotherapy may respond to a rechallenge after anti-PD-1 treatment. Targeted drugs such as the multitarget tyrosine kinase inhibitor lenvatinib, which acts on VEGFR1-3, induce immunogenic cell death (ICD) and modulate the immune response via the VEGF-VEGFR pathway (3). This action directly attacks cancer cells, mitigates immunosuppressive factors, and enhances immunotherapy efficacy (18). Therefore, the early combined use of targeted therapy, immunotherapy, and systemic chemotherapy is recommended (19).

### 4.5 Safety

Although targeted therapy and immunotherapy combined with chemotherapy may lead to more adverse reactions, these complications are generally controllable (20, 21). In our study, all patients experienced some AEs; however, no grade 5 AEs occurred. Approximately 45.6% of the patients had Grade 3 AEs, and 3.5% experienced Grade 4 AEs. Common AEs included fatigue, myelosuppression, and decreased appetite. The higher incidence of myelosuppression was attributed to chemotherapy. In comparison, adding chemotherapy to targeted therapy and immunotherapy in this study did not significantly increase the incidence of AEs (22).

### 4.6 Limitations

Although insightful, this study has several limitations, including its single-center, real-world design and small sample size, which necessitate cautious interpretation. Future research should involve larger, multicenter, prospective studies. The use of various immunotherapeutic drugs, including anti-PD-1 agents, requires further investigation through prospective, single-drug studies. Additionally, our study utilized only lenvatinib, a TKI drug. In future studies, we will explore the application of other targeted drugs in the treatment of ICC. Furthermore, the effectiveness of targeted therapy combined with immunotherapy and chemotherapy in different tumor classifications requires further confirmation; for example, we can investigate the effects of the expression of different genes on the efficacy of targeted therapies. Despite these limitations, this study offers valuable insights for future clinical research and the development of treatment strategies.

### 4.7 Conclusion

The combination of PD-1 inhibitors, TKIs, and chemotherapy is effective, safe, and tolerable for the treatment of advanced ICC. This combined treatment regimen outperforms the chemotherapy regimen alone, thereby extending the survival of patients with advanced ICC. However, further research with larger prospective cohorts is necessary to validate these findings more comprehensively.

## Data Availability

The raw data supporting the conclusions of this article will be made available by the authors, without undue reservation.
